# The evidence for natural therapeutics as potential anti-scarring agents in burn-related scarring

**DOI:** 10.1186/s41038-016-0040-1

**Published:** 2016-05-04

**Authors:** M. Mehta, O. A. Branford, K. J. Rolfe

**Affiliations:** 1 British College of Osteopathic Medicine (BCOM), Finchley Road, London, NW3 5HR UK; 2 The Royal Marsden Hospital, Fulham Rd, London, SW3 6JJ UK

**Keywords:** Burns, Hypertrophic scar, Natural therapeutics, Wound healing

## Abstract

Though survival rate following severe thermal injuries has improved, the incidence and treatment of scarring have not improved at the same speed. This review discusses the formation of scars and in particular the formation of hypertrophic scars. Further, though there is as yet no gold standard treatment for the prevention or treatment of scarring, a brief overview is included. A number of natural therapeutics have shown beneficial effects both in vivo and in vitro with the potential of becoming clinical therapeutics in the future. These natural therapeutics include both plant-based products such as resveratrol, quercetin and epigallocatechin gallate as examples and includes the non-plant-based therapeutic honey. The review also includes potential mechanism of action for the therapeutics, any recorded adverse events and current administration of the therapeutics used. This review discusses a number of potential ‘treatments’ that may reduce or even prevent scarring particularly hypertrophic scarring, which is associated with thermal injuries without compromising wound repair.

## Background

A burn is defined by the World Health Organisation (WHO) as ‘an injury to the skin or other organic tissue primarily caused by heat or due to radiation, radioactivity, electricity, friction or contact with chemicals’ [1]. It has been estimated that annually, there are 486,000 burn injuries in the USA that required medical attention with 40,000 requiring hospitalisation [2], with a global incidence in 2004 of approximately 11 million burn injuries requiring medical attention [3]. Non-fatal burns are one of the leading causes of disability in low- to middle-income countries [3]. Advances in medical treatment means that survival following extensive burns has improved over recent years though the incidence, treatment and prevention of scarring from thermal injuries has not improved over the same time frame [4].

## Review

### Hypertrophic scars

Hypertrophic scars are defined as visible raised scars which do not spread beyond the original injury margins. Hypertrophic scars are characterised by proliferation of the dermal tissue, excessive deposition of fibroblast-derived extracellular matrix (ECM) over a prolonged period of time and persistent inflammation and fibrosis [5]. Hypertrophic scars primarily contain collagen type III orientated parallel to the epidermal surface with abundant collagen nodules [6]. This structural realignment results in contracture, low tensile strength and raised scars.

The incidence of hypertrophic scars after a burn remains unclear, with estimates ranging from 26 % to 75 % depending on age, ethnicity and if healing was spontaneous or through surgical means (for example, skin grafting) [7–12].

Apart from the aesthetic problems, patients often complain of itching, redness and hard nodular scar tissue often with abnormal sensation. Hypertrophic scars following thermal injury are often associated with contractures, which can result in functional loss especially over joints such as in the hand [13].

### Scar formation

Wound healing is an inherent process which aims to restore the integrity of the skin as rapidly as possible. Wound healing is divided into four stages: haemostasis, inflammation, proliferation and tissue remodelling. Within these four stages, which often overlap, there are numerous interactions between fibrotic and anti-fibrotic growth factors, cells, ECM components and numerous enzymes [14].

**Fig. 1:**
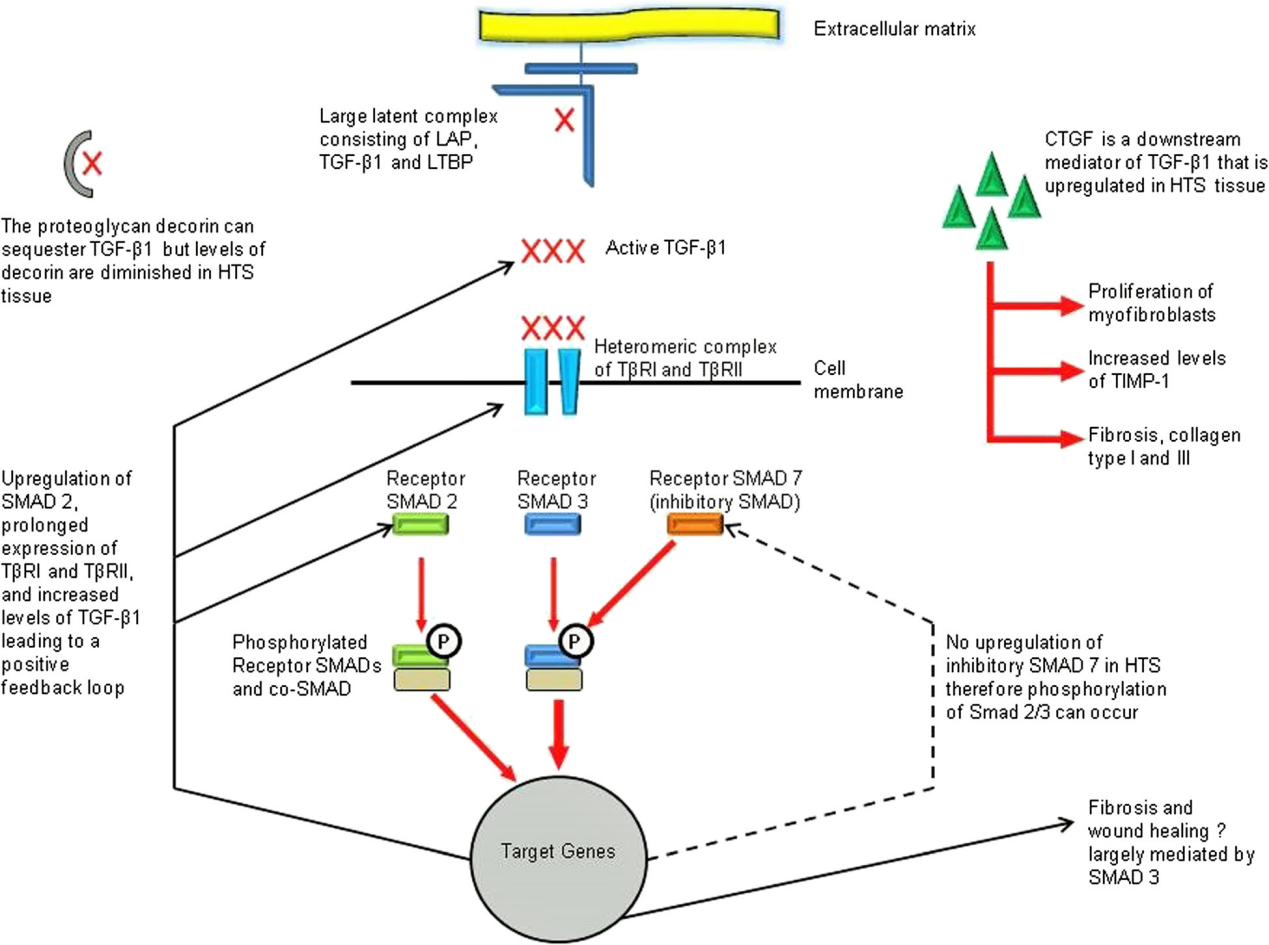
Summary of TGF-β signalling in hypertrophic scars (Reprinted from Penn JW, Grobbelaar AO, Rolfe KJ. The role of TGF-β family in wound healing, burns and scarring: a review. Int J Burns Trauma. 2012;2:18–28. With permission). *TGF-β1* transforming growth factor beta 1, *HTS* hypertrophic scar, *LAP* latency-associated peptide, *LTBP* latebt transforming growth factor-beta-1 binding portein, *CTGF* connective tissue growth factor, *TIMP-1* tissue inhibitor of metalloproteinase-1

Fibroblasts derived from hypertrophic scars have demonstrated an altered phenotype compared to fibroblasts derived from normal scars and fibroblasts derived from uninjured tissue [6, 15]. Fibroblasts derived from hypertrophic scars have demonstrated both an increased expression of the pro-fibrotic cytokine, transforming growth factor beta 1 (TGF-β1), and a prolonged expression of the associated TGF-β receptors (Fig. 1) [16, 17]. Further, there appears to be an alteration in TGF-β signalling (via increased phosphorylation of the receptor Smad proteins) in hypertrophic-derived fibroblasts and a decreased expression of the inhibitory Smad 7 in hypertrophic scar-derived fibroblasts [18]. Studies have indicated that ectopic expression of Smad 7 prevents collagen contraction in both normal and hypertrophic scar-derived fibroblasts (FPCL: fibroblast-populated collagen lattice: model for contraction) [19].

A mouse model which lacked the receptor Smad, Smad 3, showed improved wound healing [20]. Conversely, exogenous Smad 3 (via an adenovirus-containing Smad 3 cDNA) in a rabbit dermal ulcer model showed increased granulation tissue and re-epithelisation [21]. Sumiyoshi and colleagues suggested that the differences in outcome may be that the adenovirus-containing Smad targeted mainly fibroblasts [21, 22], whereas in the mouse model lacking Smad 3, the deficiency was found in fibroblasts, keratinocytes and inflammatory cells.

Decorin, a proteoglycan found in the dermal ECM, binds and regulates TGF-β1 and plays a role in collagen fibrillinogenesis. Decorin has been shown to be diminished in hypertrophic scars [23]. Zhang and his colleagues demonstrated that decorin inhibited both basal and TGF-β induced contraction in FPCL in normal and hypertrophic derived fibroblasts [24].

Linge et al. demonstrated that fibroblasts derived from hypertrophic scars failed to undergo apoptosis during FPCL contraction unlike fibroblasts derived from normal scars [25]. It was determined that the hypertrophic scar-derived fibroblasts were resistant to breakdown by collagenase D and matrix metalloproteinase-2 (MMP-2) due to excessive cross-linking of the FPCL. Linge and colleagues further found that hypertrophic scar-derived fibroblasts over-expressed tissue transglutaminase [25]. Reducing tissue transglutaminase in hypertrophic FPCL induced apoptosis on gel contraction [25]. Differences have been further identified in myofibroblasts, and these fibroblasts express alpha smooth muscle actin and are associated with wound contraction and maturation of the granulation tissue [26]. Myofibroblasts derived from hypertrophic scars appear to be less sensitive to apoptotic signals than fibroblasts derived from normal scars and express different levels of some apoptotic-related molecules [27].

Studies suggest that migrating fibrocytes, cells with a distinct cytokine and chemokine profile, may play a role in wound repair and therefore scarring [28]. Fibrocytes appear to be increased in number of healing burn wounds and were higher in hypertrophic scar than in mature scar tissue [29]. Fibrocytes from patients who have undergone thermal injury appear to differ in their paracrine effects on dermal fibroblasts by stimulating fibroblasts to proliferate, produce and contract the ECM and stimulate production of TGF-β1 and its downstream effector connective tissue growth factor (CTGF/CCN2) [30].

Matrix metalloproteinases are involved in the breakdown of the ECM during a number of physiological processes. MMP-1 is involved in the degradation of interstitial collagens, type I, II and III. Hypertrophic-derived fibroblasts appear to have reduced collagenase (MMP-1) activity [31]. Though other studies have shown an increase in expression of MMP-2 and low level of MMP-9 [32], MMP-2 has been demonstrated to effect matrix remodelling in late wound healing, degrading denatured collagen, whereas MMP-9 appears to be involved in early wound healing degrading collagen types IV and V, fibronectin and elastin [33, 34].

Evidence suggests that the immune response may play a role in scarring. Studies have suggested an abnormality in the role of Th1/Th2 paradigm after a thermal injury [30, 35]. Studies have implicated Toll-like receptors in fibrosis with recent studies implicating increased expression of toll-like receptor 4 (TLR4) mRNA and surface receptors implicating the Toll receptor system in potential activation of dermal fibroblast in hypertrophic scars [36].

### Treatment for scar

Numerous treatments are used to reduce or prevent scarring [37, 38]. Identifying injuries, which if permitted to heal spontaneously may result in pathological scarring, is important to prevent unnecessary treatment as few treatments are without side effects [39, 40]. Compression therapy (pressure garments) has shown mixed results with a meta-analysis showing no alteration in scar scores [41], whereas a 12-year prospective study showed an overall significant improvement in scar appearance [42]. The mechanism for pressure in the reduction of scarring remains unclear though in vitro studies suggest a change in MMP, collagen and alpha smooth muscle actin expression [43, 44]. Patient compliance is often low due to discomfort which may affect the overall clinical result, but further compression therapy has well-recognised complications [45, 46].

Silicone gel is commonly used in the treatment or prevention of pathological scars. Results for the use of silicone gel either on its own or with compression garments remain conflicting [47], but this may in part be due to patient compliance [48]. The mechanism of action for silicone gel remains unclear though a recent study suggests that silicone gel alters the expression of TGF-β1, platelet-derived growth factor (PDGF) and basic fibroblast growth factor (bFGF) 4 months after surgery for surgical scar revision though patient numbers were small (*n* = 4) and the original injuries were not discussed [49].

Intra-lesional corticosteroids have shown to be useful in vivo through a number of mechanisms including reduction in the inflammatory process, decrease in collagen production and fibroblast proliferation [50, 51]. Scar response rates for triamcinolone acetonide (10 to 40 mg/ml), the most common corticosteroid used for scar reduction, range from 50 % to 100 % with a recurrence rate of 9 %–53 % (reviewed in [50]). However, the use of corticosteroids is often associated with pain on injection and up to 50 % of patients report side effects [52].

Other treatments which are currently being studied include laser therapy [53], bleomycin, interferon, 5-fluorouracil, imiquimod, methotrexate and cryotherapy [54]. However, to date, there is no effective ‘gold standard’ for the treatment or prevention of any scarring.

### Plant-based products

A number of plants with medical properties have been studied for their effectiveness in the prevention of scarring [55]. The present review provides in vitro and/or in vivo evidence supporting plant-based products as potential therapeutic agents.

#### Quercetin

Quercetin is a flavonoid found in plants, vegetables and fruits including onions, apples and berries [56]. Quercetin has been demonstrated in vitro to have a number of biological properties including tumour suppression and anti-inflammatory, anti-oxidant properties and is anti-bacterial [57–60]. However, the metabolism of quercetin in humans may reduce its biological effects [61].

Quercetin has been shown in vitro to reduce proliferation in fibroblasts derived from keloid scars and alter intracellular signalling pathways and collagen synthesis [62–64]. Phan and colleagues demonstrated that in fibroblasts derived from keloid and hypertrophic scars, quercetin not only inhibited fibroblast proliferation by inducing cell cycle arrest but also inhibited FPCL contraction, though both cell cycle arrest and FPCL could be reversed and though resumption of contraction was slowest in the quercetin treated group [65]. Saulis and colleagues showed in a rabbit model that Mederma (active compound allium cepa, a derivative of quercetin) improved collagen organisation and therefore may have an effect on the pathophysiology of hypertrophic scars [66].

#### Onion extract

Onion extract in in vitro studies suggest that it may have anti-inflammatory and anti-proliferative properties on fibroblasts and mast cells and increase the expression of MMP-1 [67, 68]. Quercetin and onion extract have both been shown to induce the up-regulation of MMP-1 in vitro and in vivo [68]. MMP-1 is known to play a role in ECM remodelling and therefore quercetin and onion extract may play a role in anti-fibrotic processes.

A small (*n* = 16) randomised controlled split scar study on Asian women undergoing a Pfannenstiel’s incision for caesarean section demonstrated a statistically significant reduction in scar height and symptoms at 4 and 12 weeks post-surgery in an onion extract group. However, there was no statistically significant reduction in redness or pliability of the scar over the time studied [69]. Ho et al. using a gel containing onion extract, heparin and allotonin found the gel significantly reduced the risk of scarring in 120 Chinese patients undergoing laser removal of their tattoos [70]. Wananukul et al., in a paediatric group (*n* = 39; mean age 4.3 years old) who underwent a median sternotomy in a split scar experimental study (onion extract versus placebo), demonstrated that onion extract in a silicone derivative gel significantly decreased the incidence of hypertrophic scars, whereas there was no significant difference in the incidence of keloid scars [71]. Other authors have used a combination of a silicone derivative plus onion extract in patients who had undergone a median sternotomy (*n* = 60) over a treatment period of 12 weeks. They found that itch and pain was less for the treated group, there was also an improved Vancouver Scar score in the treated group especially for pigmentation [72].

Beuth and colleagues compared hypertrophic scars treated with Contractubex® (cepae extract, heparin, allantoin; treatment group) for 28 days with one intra-lesional corticosteroid application (control group) [73]. Contractubex® demonstrated a significant shorter time for normalisation of the scar (erythema, pruritus and consistency) compared to the corticosteroid group. Contractubex® was further associated with less adverse events than corticosteroid application [73].

#### Resveratrol

Resveratrol is a natural plant polyphenol and phyto-oestrogen, present in grape skin, red wine, and peanuts [74, 75]. Resveratrol is noted to have a number of beneficial health effects including cardio-vascular, anti-inflammatory and anti-oxidant properties [74–78].

Resveratrol has been shown to reduce fibroblast cell proliferation through cell cycle arrest at G1 in fibroblasts derived from hypertrophic scars and normal skin fibroblasts and induce apoptosis [79]. Resveratrol further decreased hydroxyproline levels and down-regulated the expression of collagen type I and III mRNA [79].

Resveratrol has further shown beneficial effects in preventing surgical adhesions in an animal model [80]. Ikeda et al. demonstrated in vitro that resveratrol decreases TGF-β1, type 1 collagen and alpha smooth muscle actin in keloid-derived fibroblasts [81]. Further, resveratrol suppressed keloid-derived fibroblast proliferation and induced apoptosis. Interestingly, resveratrol did not have the same effects on alpha smooth muscle actin or type 1 collagen in fibroblasts derived from normal scars [81].

#### Epigallocatechin gallate (EGCG)

EGCG is a major catechin in green tea and has a number of biological properties; it has been shown to potentially play a role in preventing fibrosis in a number of organs [82].

EGCG has been shown in FPCL to abrogate contraction stimulated by PDGF and TGF-β1 [83, 84]. EGCG binds directly to PDGF-BB preventing the PDGF ligand binding to its receptor and therefore preventing both proliferation and FPCL contraction [83, 85]. EGCG has been shown to inhibit a number of intracellular signalling pathways and reduce expression of pro-fibrotic molecules (vascular endothelial growth factor (VEGF), TGF-β1, CTGF) in a number of organs [86–88]. Inhibition of TGF-β1 results in reduction of the synthesis of the ECM [84]. Interestingly, EGCG has been demonstrated to improve re-epithelisation in a chronic wound model and the structural stability of collagen was shown to be enhanced with EGCG [89, 90].

#### Oleanolic acid (OA)

OA is a naturally occurring triterpenoid compound with a number of biological properties including anti-inflammatory and anti-tumour effects [91, 92]. In a rabbit ear model of hypertrophic scarring where OA was applied daily for 22 days, it was found to significantly inhibit hypertrophic scarring with a corresponding reduction in TGF-β1 and collagen type I and III and increase levels of MMP-1 [93]. Zhang et al. also used the rabbit ear model to study OA and repeated the observation that OA reduced the incidence of hypertrophic type scars [94]. They found that TGF-β1, MMP-1, TIMP-1 and collagen I and III were notably decreased though the number of apoptotic cells and mRNA expression of MMP-2, caspase-3 and caspase-9 were increased in the scar tissue [94].

#### Curcumin

Curcumin, a polyphenol, has been shown to induce apoptosis in a number of cell lines [95–97]. Curcumin has been shown in a rat wound healing model to increase contraction and reduce wound healing time [98]. The wounds showed increased fibronectin and collagen expression with increased collagen maturation and cross-linking increasing the wounds tensile strength after treating with curcumin for 12 days (200 μl at a concentration of 40 mg/kg body weight) [98].

Scharstuhl and colleagues showed that curcumin treatment (>25 μM for 48 h) induced fibroblast apoptosis and inhibited FPCL contraction via a reactive oxygen species (ROS)-mediated process in human dermal fibroblasts in vitro [99]. They concluded that curcumin at high concentrations may be a therapeutic strategy in the reduction or prevention of hypertrophic scarring and that the process can be regulated through the modulation of heme oxygenase(HO) molecule activity or the administration of HO effector molecules.

#### Shikonin

Shikonin is a natural naphthoquinone compound from the Chinese herb *Lithospermum erythrorhizon*. Shikonin has been demonstrated to have a number of molecular targets, inducing apoptosis, necrosis and necroptosis in cancer cells [100–102]. It has further been demonstrated that shikonin selectively kills cancer cells while maintaining normal cells [103]. Shikonin in cancer lines has been shown to alter a number of intracellular signalling pathways particularly those associated with apoptosis [103–105]. Fan and colleagues demonstrated that Shikonin keratinocytes did not respond to Shikonin unlike human scar-derived fibroblasts which where stimulated to undergo apoptosis [106]. Shikonin induced apoptosis by altering the expression of capsase-3, B-cell lymphoma (BCL)-2, phosphorylation of ERK1/2 and p38 [107]. Further, Shikonin down-regulates collagen (type I and III) and smooth muscle actin gene expression in scar-derived fibroblasts [107].

Normal skin fibroblasts (*n* = 3) were demonstrated to reduce TGF-β1 induced collagen production when cultured with Shikonin. This was demonstrated to be through alteration of the TGF-β1-SMAD intracellular signalling pathway [108]. This pathway further prevented FPCL by down-regulating alpha smooth muscle actin [108].

#### Emodin

Emodin is a resin derived from the Himalayan rhubarb, buckthorn and Japanese knotweed. It has been investigated for a number of therapeutic effects including asthma, arthritis and Alzheimer’s disease in a number of animal models [109–112]. Emodin has been shown to alter a number of intracellular signalling pathways including nuclear factor-κB and phosphoinositide 3 kinase/Akt [113], which plays a role in a number of cellular processes including the cell cycle. In vitro and in vivo studies have suggested that emodin may potentially play a role in preventing fibrosis in a number of organs [113–116].

Hypertrophic scars were developed through mechanical stress in an animal model, and emodin was administered intra peritoneally (10 mg/kg). Liu demonstrated that the emodin-treated hypertrophic scar group had an improved histopathological appearance compared to the control group; however, on removal of emodin at day 14, histopathology of the scar was only minimally improved at day 28 [113]. Emodin further inhibited the inflammatory response in the hypertrophic scars (tumor necrosis factor (TNF)-α monocyte chemoattractant protein (MCP)-1, interleukin (IL)-6). Emodin was shown to reduce the activation of PI3K and Akt in the hypertrophic fibroblasts, but this was not reciprocated in normal fibroblasts [113].

### Non-plant-based therapeutics

#### Honey

Honey has been shown to have anti-bacterial properties through the presence of inhibines which consist of hydrogen peroxide, flavonoids, phenolic acids and other as yet unidentified substances [117, 118]. Other non-peroxide anti-microbial factors have been identified in honey depending on the floral sources, origin and processing [119–123]. However, studies have implicated that it is not simply its anti-microbial properties that confer its effectiveness in treating wounds [124]. Honey activates various components of the immune system in vitro and in vivo which not only activates the immune response but also tissue repair [125–129].

To date, there have been mixed results with the use of honey on wounds. Nakajima and colleagues using a mouse model and three forms of Japanese honey found that the use of honey had little benefit in wound healing [130]. Gupta and colleagues retrospectively compared the hospital records of burns patients who had been treated with either honey dressings or silver sulfadiazine dressings over a period of 5 years [131]. They found that honey enhanced healing, reduced contractures and had better overall outcome compared to silver sulfadazine [131]. Others have confirmed the beneficial effects of honey and healing time when compared to other dressings including silver sulfadazine-, film- and gauze-based dressings [132, 133]. However, silver sulfadiazine has been shown to delay healing and increase pain and infection rates and may therefore have not been the best comparator [134]. Honey’s anti-inflammatory effect is proposed as the reason why honey reduces fibrosis and scarring [135–137].

### Adverse events, bioavailability interactions and synergistic effects

Though considered ‘natural’, most of the products are synthetically manufactured; further, even some ‘natural’ products have been identified as causing toxicities (Table 1) [138, 139]. There have been limited toxicity studies conducted on the natural therapeutics discussed in this review, though those used in human studies appear to have mild adverse events recorded (such as honey, onion extract, quercetin; Table 1). Though there have been individuals who appear to have increased adverse events, resveratrol saw one individual in a study show grade 4 elevation of their liver function markers after 3 months treatment of 1 g of resveratrol daily [140]. The patient’s markers returned to normal after discontinuing the medication. EGCG has also shown in some individuals to elevate liver function tests though one study concluded it was an issue with the lot [141], though a case study did identify drug-induced hepatitis with the use of a concentrated green tea extract [142]. Oleanolic acid in animal studies suggests that repeated oral administration can cause liver injury [138]. Oleanolic acid derivatives have been also shown to be related to fluid overload which in some individuals resulted in heart failure in patients with stage 4 chronic renal disease (8.8 % of the treated group compared to 5 % of the placebo group) [139].

**Table 1:** Natural therapeutics, where they originate from, their potential mechanism of action and known adverse events, bioavailability and drug interactions

Natural therapeutic agent	Origin	Mechanism of action(s)	Administered	Known adverse effects or potential issue with use
Quercetin	Flavonoid found in plants, vegetables and fruits	• Blocks TGF-β (inhibits receptor expression and SMAD2/3 nuclear translocation)—in turn alters collagen expression [62] • Alters IGF-1 signalling (through reduction in receptor and intracellular signalling)—in turn affects keloid fibroblast proliferation [63] • Reduces collagen contraction [65]	• In vitro [62, 63, 65]	• Bioavailability is problematic though studies have suggested potential ways to improve its availability [152] • Adverse events appear mild [153, 154] • Interacts with some drugs, e.g. fluoroquinolones, taxol/paciltaxel [144, 145]
Onion extract (kaempferol, Mederma®, Contractubex®, Cybele®, Erasé gel, Kaloidon gel)	Onion	• Up-regulates MMP-1 [68]	• In vitro (human skin fibroblasts) [68] • In vivo (hairless mice administered with ointment) [68]	• No adverse events [69, 72] • Moderate pruritus, all other adverse events less than the use of corticosteroids [73]
Resveratrol	Grape skin, red wine and peanuts	• Inhibits fibroblast cell growth, causes cell cycle arrest and induces apoptosis which result in reduced collagen expression [79] • Reduced TGF-β1 protein in keloid fibroblasts (*n* = 5), reduced cell proliferation and induced apoptosis but did not decrease collagen type I, alpha smooth muscle actin or heat shock protein 47 in normal skin fibroblasts (*n* = 1) [81]	• In vitro (hypertrophic-derived fibroblasts, normal skin fibroblasts) [79] • In vitro (keloid fibroblasts) [81]	• In vitro appears to have no genotoxic activity [155] • Resveratrol (administered orally) in a number of studies in humans both symptomatic (e.g. Alzheimer’s patients, obese patients) and healthy showed minor adverse events, the most common being nausea, weight loss, diarrhoea and skin rash [140, 156, 157] • One individual showed elevated hepatic ALT and AST (grade 4) which returned to normal after stopping the medication [140] • Boocock et al. [149] suggested oral administration may not be sufficient for some therapeutic roles of resveratrol
Epigallocatechin gallate	Green tea	• Prevents PDGF-BB binding to its receptor and leads to prevention of proliferation and collagen gel contraction [83, 85] • Known to inhibit a number of intracellular signalling pathways and thereby reducing pro-fibrotic gene expression [86–88] and ECM production [84]	• In vitro (neonatal fibroblasts) [83] • In vitro (human/rat vascular smooth muscle cells) [85] • In vitro (post-natal human dermal fibroblasts [84] • In vitro (rat cardiac fibroblasts) [86] • In vitro (human gingival fibroblasts) [87] • In vitro (human umbilical vein endothelial cells) [88]	• EGCG appears well tolerated with oral administration [158–160] or used on the skin [161] • Adverse events include mild gastrointestinal issues and skin rashes [158, 160, 161] • Polyphenon E has been linked to elevated liver function tests but this appeared related to the LOT [141] though a case study showed a case of drug-induced hepatitis [142] and other studies have shown minor increase in liver markers [162] • Number of chemotherapy agents [146]
Oleanolic acid	Number of foods, for example, olive oil, garlic, etc	• Decreased TGF-β1 and collagen I and III and increased MMP-1 [93] possibly through decreased fibroblast proliferation, increased apoptosis and degradation of collagen types I and III through enhanced MMP-2 activity [94]	• In vivo (rabbit ear model for hypertrophic scars; applied as an ointment) [93, 94]	• Animal model associated with male infertility [163] • Oral administration in an animal model (dose, 22.5–135 mg/kg) for 5 days. Liver injury observed at doses of 90 mg/kg and above [138] • Bardoxolone methyl—semi-synthetic triterpenoid based on the scaffold of oleanolic acid—caused heart failure in patients with stage 4 chronic kidney disease [139]
Curcumin	Rhizome of *Curcuma longa* and related species.	• Induced fibroblast apoptosis and reduced collagen gel contraction [99] via ROS mechanism	• In vitro (human fibroblasts) [99]	• Poor bioavailability especially after oral administration [164] • Appears well tolerated up to 8 g/day up to 3 months [164, 165] • Adverse effects may change with adaptations that are used to improve bioavailability • Chelate iron suppresses hepcidin therefore potentially causing iron deficiency [166] • Interacts with 5-fluorouracil and vinorelbine [140, 147, 148, 156]
Shikonin	Chinese herb Radix Arnebiae	• Induces apoptosis in fibroblasts [106] • Down-regulates collagen types I and III and α smooth muscle actin [107] • Appears to induce apoptosis by altering p-ERK 1/2, p-p38 and caspase-3 [107]	• In vitro (human keratinocytes, skin fibroblasts) [106] • In vitro (human keratinocytes, human skin fibroblasts, hypertrophic scar-derived fibroblasts) [107]	• Low bioavailability due to high lipophilicity [167] altered through the formation of a complex with other proteins [150] • Limited toxicity studies—one animal study demonstrated that it appeared safe up to concentrations of 800 mg/kg for 180 days [168]
Emodin	Derived from the Himalayan rhubarb, buckthorn and Japanese knotweed	• Alters the intracellular pathway of Pi3K and Akt but only in hypertrophic scar-derived fibroblasts [113] and this in turn inhibited the inflammatory response and improved the histopathology appearance of the scar [113]	• In vivo and in vitro (mice model for hypertrophic scars, emodin was administered intra-peritoneally; mice derived hypertrophic scarring fibroblasts and normal fibroblasts) [113]	• Not known as yet
Honey		• Accelerates wound healing due to its anti- bacterial activity, anti-oxidant activity, stimulator effects and anti-inflammatory effects [135–137]	• Human patients with burns—honey-impregnated gauze [135, 136]	• Stinging pain on administration, local atopic reactions in paediatric group [169]

*TGF-β1* transforming growth factor beta 1, *IGF-1* insulinlike growth factor-1, *MMP* matrix metalloproteinase, *PDGF-BB* platelet-derived growth factor-BB, *ECM* extracellular matrix, *RGCG* epigallocatechin gallate, *ROS* reactive oxygen species

It has been well recognised that some herbal products can interact with medicinal drugs and reduce or prevent their effectiveness, e.g. St John’s wort (*Hypericum perforatum*), and in some cases, alter the efficacy of medicinal drugs [143]. A number of the products discussed in this paper have also been shown to interact with other drugs including antibiotics (fluoroquinones) and chemotherapy agents [144–148].

A number of the agents have been shown to have low bioavailability (quercetin, curcumin, shikonin), and others have been suggested that oral administration may not be sufficient for therapeutic levels to be reached or indeed maintained [149]. Further, those that have low bioavailability which are then either manipulated or other proteins added this structural alteration may affect both adverse events and the actual therapeutic mechanisms [150, 151]. To date, there remains a paucity of information in regard to the safety of some of these agents in their use as anti-scarring products.

## Conclusions

In vitro and in vivo studies have shown that a number of ‘natural’ therapeutic agent and strategies may play a role in the future treatment of scarring, particularly hypertrophic scarring which is so intrinsically linked with burn injuries. There remains no gold standard in the treatment or prevention of scarring. It remains problematic comparing all products not just natural therapeutics in part due to the number of methodologies used to assess the effectiveness of anti-scarring therapeutics and the number of models used. Further, those that do undergo clinical trials, the variation in patients and outcome measures is immense leading to problems in comparing agents and is often undertaken once the scar has formed. There is a theoretical risk which agents that reduce or prevent scarring may in turn prevent or lengthen the wound healing process, and this has yet to be elucidated. However, it appears that there is a potential for a natural therapeutic as either a monotherapy or as an adjunct to play a role in treating or even preventing hypertrophic scarring.

## Abbreviations

ECM: extracellular matrix
FPCL: fibroblast-populated collagen lattice
MMP: matrix metalloproteinase
